# Central effects of peripherally administrated immune cells modulated by a psychoactive substance in aggression

**DOI:** 10.1192/j.eurpsy.2024.1421

**Published:** 2024-08-27

**Authors:** E. Markova, E. Serenko, M. Knyazheva, A. Akopyan, M. Tikhonova, T. Amstislavskaya

**Affiliations:** ^1^Neuroimmunology Lab, State Research Institute of Fundamental and Clinical Immunology; ^2^State Research Institute of Neurosciences and Medicine, Novosibirsk, Russian Federation

## Abstract

**Introduction:**

It is known that the formation of aggressive behavior is accompanied by neurodegenerative and neuroinflammatory changes. Immune cells have a regulatory effect on the central nervous system functions, including regulation of behavior.

**Objectives:**

We first demonstrated that *ex vivo* chlorpromazine - modulated immune cells have a positive aggressive behavior editing effect. The aim of the study was to investigate the influence of the indicated cells on some central mechanisms underlying the development of aggressive reactions.

**Methods:**

(CBAxC57Bl/6) F1 aggressive male mice, developed in conditions of chronic social stress, were undergoing the transplantation of syngeneic spleen lymphocytes with *ex vivo* chlorpromazine-modulated functional activity. In recipients the immunohistochemical analysis was performed assessing the expression of the microglial marker Iba1. The levels of brain-derived neurotrophic factor (Bdnf ) and cytokines was assessed by ELISA. For histological examination Nissl staining was applied.

**Results:**

Aggressive behavior editing after the chlorpromazine-modulated immune cells transplantation registered against the background of some structural and functional changes in the brain. It was found an increase in the density of pyramidal neurons in CA1 and CA3 hippocampal regions and augmented level of Bdnf. The decreased expression of microglial activation marker Iba1, accompanied with decreased levels of pro-inflammatory cytokines (IL-1β, IL-2, IL-6, INF-γ) and increased  anti-inflammatory (IL-4) cytokine was found. Visualization of functionally active lymphocytes pre-treated with chlorpromazine in the brain parenchyma of aggressive recipients suggests a direct effect of injected lymphocytes on CNS.

**Conclusions:**

The effect of chlorpromazine - modulated immune cells that edits aggressive behavior is realized by stimulating neurogenesis, neuroplasticity and reducing neuroinflammation.

**Disclosure of Interest:**

None Declared

